# GPR30 Activation by 17β-Estradiol Promotes p62 Phosphorylation and Increases Estrogen Receptor α Protein Expression by Inducing Its Release from a Complex Formed with KEAP1

**DOI:** 10.3390/jpm11090906

**Published:** 2021-09-11

**Authors:** Chia-Lung Tsai, Chiao-Yun Lin, Angel Chao, Yun-Shien Lee, Ren-Chin Wu, Chi-Neu Tsai, Chih-Feng Yen, An-Shine Chao

**Affiliations:** 1 Genomic Medicine Research Core Laboratory, Linkou Chang Gung Memorial Hospital, Taoyuan 333011, Taiwan; cltsai24@gmail.com (C.-L.T.); bojack@mail.mcu.edu.tw (Y.-S.L.); 2 Department of Obstetrics and Gynecology, Linkou Chang Gung Memorial Hospital and Chang Gung University College of Medicine, Taoyuan 333423, Taiwan; chiao.yun0101@gmail.com (C.-Y.L.); drangiechao@gmail.com (A.C.); yen2158@cgmh.org.tw (C.-F.Y.); 3 Gynecologic Cancer Research Center, Linkou Chang Gung Memorial Hospital, Taoyuan 333423, Taiwan; 4 Department of Biotechnology, Ming-Chuan University, Taoyuan 33348, Taiwan; 5 Department of Pathology, Linkou Chang Gung Memorial Hospital, Taoyuan 333423, Taiwan; renchin.wu@gmail.com; 6 Graduate Institute of Clinical Medical Sciences, Chang-Gung University, Taoyuan 33302, Taiwan; pink7@mail.cgu.edu.tw; 7 Department of Obstetrics and Gynecology, New Taipei Municipal Tu Cheng Hospital, New Taipei City 236012, Taiwan

**Keywords:** p62, ESR1, KEAP1, endometrial cells

## Abstract

Estrogens can elicit rapid cellular responses via the G-protein-coupled receptor 30 (GPR30), followed by estrogen receptor α (ERα/ESR1)-mediated genomic effects. Here, we investigated whether rapid estrogen signaling via GRP30 may affect ESR1 expression, and we examined the underlying molecular mechanisms. The exposure of human endometrial cancer cells to 17β-estradiol promoted p62 phosphorylation and increased ESR1 protein expression. However, both a GPR30 antagonist and GPR30 silencing abrogated this phenomenon. GPR30 activation by 17β-estradiol elicited the SRC/EGFR/PI3K/Akt/mTOR signaling pathway. Intriguingly, unphosphorylated p62 and ESR1 were found to form an intracellular complex with the substrate adaptor protein KEAP1. Upon phosphorylation, p62 promoted ESR1 release from the complex, to increase its protein expression. Given the critical role played by p62 in autophagy, we also examined how this process affected ESR1 expression. The activation of autophagy by everolimus decreased ESR1 by promoting p62 degradation, whereas autophagy inhibition with chloroquine increased ESR1 expression. The treatment of female C57BL/6 mice with the autophagy inhibitor hydroxychloroquine—which promotes p62 expression—increased both phosphorylated p62 and ESR1 expression in uterine epithelial cells. Collectively, our results indicate that 17β-estradiol-mediated GPR30 activation elicits the SRC/EGFR/PI3K/Akt/mTOR signaling pathway and promotes p62 phosphorylation. In turn, phosphorylated p62 increased ESR1 expression by inducing its release from complexes that included KEAP1. Our findings may lead to novel pharmacological strategies aimed at decreasing ESR1 expression in estrogen-sensitive cells.

## 1. Introduction

While estrogens play key roles in the female reproductive system and fertility, abnormal exposure has long been considered a significant risk factor for endometrial and breast malignancies [1]. Estrogens are known to exert canonical genomic effects that occur mainly via the activation of estrogen receptor α (ESR1) [2]. Upon binding of estrogens, ESR1 translocates to the cell nucleus, where it binds to prototypical estrogen response elements on specific genes to alter gene expression. In the meantime, estrogens are also capable of inducing rapid alterations in signaling processes [3], such as effects mediated by specific interactions with the G-protein-coupled receptor 30 (GPR30), to activate the PI3K/AKT and EGFR/MAPK signaling pathways [4]. Interestingly, various reports showed that estrogen-induced GPR30 activation is involved in breast [5] and endometrial [6] tumorigenesis. Other studies have also demonstrated an adverse prognostic impact of GRP30 overexpression in gynecological malignancies [7,8]. While both conventional effects of estrogens are well attested, evidence to support the idea that rapid signaling via GRP30 possesses the ability to modulate canonical estrogen genomic signaling, by influencing ESR1 expression, remains limited.

Among GPR30-mediated signaling effects, estrogens can activate the p38 pathway to promote autophagy—which is vital for protein control and cellular homeostasis [9] Interestingly, the multifunctional protein p62—which is widely used as a protein marker, whose expression is negatively correlated with autophagy activation—has recently emerged as a key mediator of the association between estrogens and autophagy [10]. Multiple functional domains of p62 have been described [10,11]—including the N-terminal Phox1 and Bem1p (PB1) domain, the zinc finger (ZZ) domain, the tumor necrosis factor-associated factor 6 binding motif (TB), the LC3 interaction region (LIR), and the ubiquitin-associated domain (UBA). Because of its peculiar properties, p62 may operate as an adaptor protein to transport misfolded and ubiquitinated proteins for autophagic and proteasomal degradation. In addition, p62 plays a significant role in cell signaling, by directly binding to KEAP1, which functions as a substrate adaptor protein and a stress sensor [12]. Interestingly, the phosphorylation of serine 349 (S349) in p62 has been shown to increase the binding affinity to KEAP1 [13].

The aim of this study was two-fold. First, we investigated whether rapid estrogen signaling via GRP30 may affect their canonical genomic effects by regulating ESR1 expression. Second, we sought to shed light on the molecular underpinnings of 17β-estradiol signaling through GPR30—with a special focus on the roles played by p62 and KEAP1 as potential mediators. Knowledge of this complex interplay may pave the way for novel signaling- or autophagy-mediated pharmacological strategies aimed at decreasing ESR1 expression in estrogen-sensitive cells.

## 2. Materials and Methods

### 2.1. Cell Culture and Treatment of Cell Lines

Human breast cancer cells (MCF7, T47D) and kidney epithelial 293 cells were obtained from the American Type Culture Collection (Manassas, VA, USA). Human endometrial cancer cells (Ishikawa cells) were kindly provided by Dr. Masato Nishida (Kasumigaura Medical Center, Ibaraki, Japan) [14]. MCF7, T47D, and 293 cells were cultured in DMEM/F12 with 10% fetal bovine serum and 1% penicillin and streptomycin. Ishikawa cell was maintained in alpha-MEM medium with 15% fetal bovine serum and 1% penicillin and streptomycin. To explore the impact of the pharmacological modulation of different signaling pathways after exposure to 17β-estradiol, cells were pretreated with a proteasome inhibitor (MG132; Sigma-Aldrich, St. Louis, MO, USA; concentration: 5 μM), a protein synthesis inhibitor (cycloheximide; Sigma-Aldrich; concentration: 25 μM), a GRP30 agonist (G1; Tocris Bioscience, Bristol, UK; concentration: 100 nM), a GRP30 antagonist (G15; Tocris Bioscience, concentration: 100 nM), 17β-estradiol (Sigma-Aldrich; concentration: 100 nM), a PI3K inhibitor (LY294002; Sigma-Aldrich; concentration: 1 μM), an EGFR inhibitor (lapatinib; GlaxoSmithKline plc, London, UK, concentration: 120 nM), an SRC inhibitor (Sigma-Aldrich; concentration: 1 μM), an mTOR inhibitor that activates autophagy (everolimus; Novartis Pharma AG, Basel, Switzerland; concentration: 1 μM), a PKC inhibitor (sotrastaurin; MedChemExpress; concentration: 1 μM), a TAK inhibitor (takinib; MedChemExpress; concentration: 1 μM), a casein kinase inhibitor (PF-670462; MedChemExpress; concentration: 1 μM), and an autophagy inhibitor (chloroquine; Sigma-Aldrich; concentration: 10 μM).

### 2.2. Isolation of Primary Endometrial Stroma Cells

Primary endometrial stromal cells were isolated and maintained as previously described [15]. In brief, endometrial tissue was minced and digested in Hanks’ balanced salt solution (Invitrogen, Carlsbad, CA, USA) with collagenase B (15 U/mL; Roche, Basel, Switzerland), deoxyribonuclease I (150 U/mL, Roche) and penicillin/streptomycin at 37 °C for 60 min under agitation. Digested tissue was passed through 40 μm cell sieves (BD, Franklin Lakes, NJ, USA) and isolated primary endometrial stroma cells were cultured in DMEM/F12 with 10% fetal bovine serum.

### 2.3. DNA Construction

Full-length and truncated ESR1 and KEAP1 constructs were amplified from myc-ESR1 [16] or KEAP1 cDNA (Sino biological, Beijing, China) and recombined into the pNTAP expression vector (ESR1; Agilent Technologies, Santa Clara, CA, USA) or pCMV-3xFLAG tag vector (KEAP1, Agilent Technologies) using an In-Fusion HD cloning kit (Clontech, Mountain View, CA, USA). HA-p62 constructs have been described previously [17]. HA-p62 S349D and HA-p62 S349A were generated using overlap extension PCR with a Q5 site-directed mutagenesis kit (New England Biolabs, Ipswich, MA, USA). The primers used to generate DNA constructs are reported in Table S1.

### 2.4. DNA and siRNA Transfection

Two hundred and ninety-three cells were seeded (concentration: 5 × 10^6^ cells) in a 10 cm dish and transfected with DNA plasmids (5 μg) using the GenJet™ reagent (SignaGen Laboratories, Frederick, MD, USA). Ishikawa cells, T47D, MCF7, and primary uterine stroma cells were seeded (concentration: 1 × 10^6^ cells) in a 6 cm dish and transfected with double-stranded RNA (50 nM) using the Lipofectamine RNAiMAX reagent (Invitrogen). P62 and KEAP1 small interfering (si)RNAs were obtained from Santa Cruz Biotechnology (Dallas, TX, USA), whereas GPR30 siRNA was from Invitrogen. At 72 h after transfection, the silencing of target genes was confirmed by Western blot.

### 2.5. Cell Proliferation Assay

For BrdU assay, approximately 10,000 cancer cells were seeded in a 96-well plate overnight. After siRNA transfection for 48 h, cells were treated with 17β-estradiol or vehicle control for an additional 24 h. BrdU was added for 2 h before assessing DNA synthesis using a commercially available kit (Roche). The same siRNA transfection procedure and exposure to 17β-estradiol was used for Ki67 immunochemistry staining. To this aim, cancer cells were cultured on a six-well plate with a cover glass. The expression levels of endogenous Ki67 (cell signaling) were determined as previously described [18].

### 2.6. Western Blot

The expression levels of proteins of interest were analyzed with Western blot as previously described [19]. In brief, cells were lysed in RIPA buffer (150 mM NaCl, 20 mM Tris-Cl pH 7.5, 1% Triton X-100, 1% NP40, 0.1% SDS, and 0.5% deoxycholate) with the addition of proteinase and phosphatase inhibitors (Bionovas, Toronto, ON, Canada). Lysates were subjected to SDS-PAGE and separated proteins were subsequently transferred onto nitrocellulose membranes. The following antibodies were used: KEAP1 (Abclonal, Woburn, MA, USA), ESR1 (Abcam, Cambridge, UK), p62 (GeneTex, Hsinchu, Taiwan), phosphorylated (Ser349) p62 (Cell Signaling Technology, Danvers, MA, USA), LC3 (Abcam), HA tag (Cell Signaling Technology), FLAG tag (M2 antibody, Sigma-Aldrich), CBP tag (pNTAP expression vector; Agilent), ubiquitin (Cell Signaling Technology), GPR30 (Abclonal), and GAPDH (Santa Cruz Biotechnology). The corresponding horseradish peroxidase-conjugated antibodies were obtained from Santa Cruz Biotechnology, whereas chemiluminescence reagents were from Millipore. The signal intensity of autoradiograms was quantified with the ImageJ software (https://imagej.nih.gov/ij/; accessed on 8 January 2020) after normalization to the corresponding GAPDH intensity.

### 2.7. Immunoprecipitation

Cells were lysed in cell lysis buffer (20 mM Tris-Cl pH 7.4, 25 mM NaCl and 0.1% NP40) containing proteinase inhibitors and subsequently subjected to an overnight incubation with streptavidin beads (Invitrogen) for the pNTAP vector or the anti-KEAP1 antibodies at 4 °C under agitation. After three washes with a buffer (20 mM Tris-Cl pH 7.4, and 25 mM NaCl), pulled-down complexes were subjected to electrophoresis and detected with specific antibodies.

### 2.8. Confocal Microscopy

Ishikawa cells were seeded (concentration: 2 × 10^5^ cells) overnight in a cover slip glass, fixed with cold acetone for 20 min at 4 °C, and incubated in blocking buffer (Thermo Fisher Scientific, Waltham, MA, USA) for 1 h at room temperature. Detection of endogenous proteins was performed by incubating cells with antibodies raised against ESR1, KEAP1, p62, and control IgG (Santa Cruz Biotechnology) followed by exposure to the corresponding fluorescent antibodies (Alexa Fluor 488 dye or Alexa Fluor 546 dye; ThermoFisher). Finally, slides were examined on a Leica TCS SP2 confocal laser scanning microscope (Leica Microsystems GmbH, Wetzlar, Germany).

### 2.9. Proximity Ligation Assay

Proximity ligation assay was performed using the Duolink in situ starter kit (Sigma-Aldrich) according to manufacturer’s protocol. Briefly, deparaffinized human endometrial specimens were stained using the following antibodies: ESR1 and KEAP1 antibodies, ESR1 and p62 antibodies, and control IgG. Slides were examined using the Leica TCS SP2 confocal laser scanning microscope.

### 2.10. Animal Experiments and Immunohistochemistry

All animal experiments were reviewed and approved by the Institutional Animal Care and Use Committee of the Chang Gung Memorial Hospital (approval number: 2018120504). Five-week-old female C57BL/6 mice were treated with the autophagy inhibitor hydroxychloroquine (Sanofi, Paris, France) at a dose 100 mg/kg or a vehicle for five days a week (total treatment duration: five weeks). Thereafter, mice were sacrificed and the paraffin-embedded uterine tissues were sectioned at 4 μm thickness and deparaffinized with xylene. Sections were dehydrated through a series of graded ethanol baths and stained with antibodies raised against ESR1 (Abcam), phosphorylated p62 (Cell signaling), and KEAP1 (Abclonal) in an automated immunohistochemical stainer (Leica bond polymer refine detection kit; Buffalo Grove, IL, USA) according to the manufacturer’s protocol. Hematoxylin was used for counterstaining.

## 3. Results

### 3.1. ESR1, KEAP1, and p62 Interact with Each Other and Are Capable of Forming a Complex

**Figure 1:**
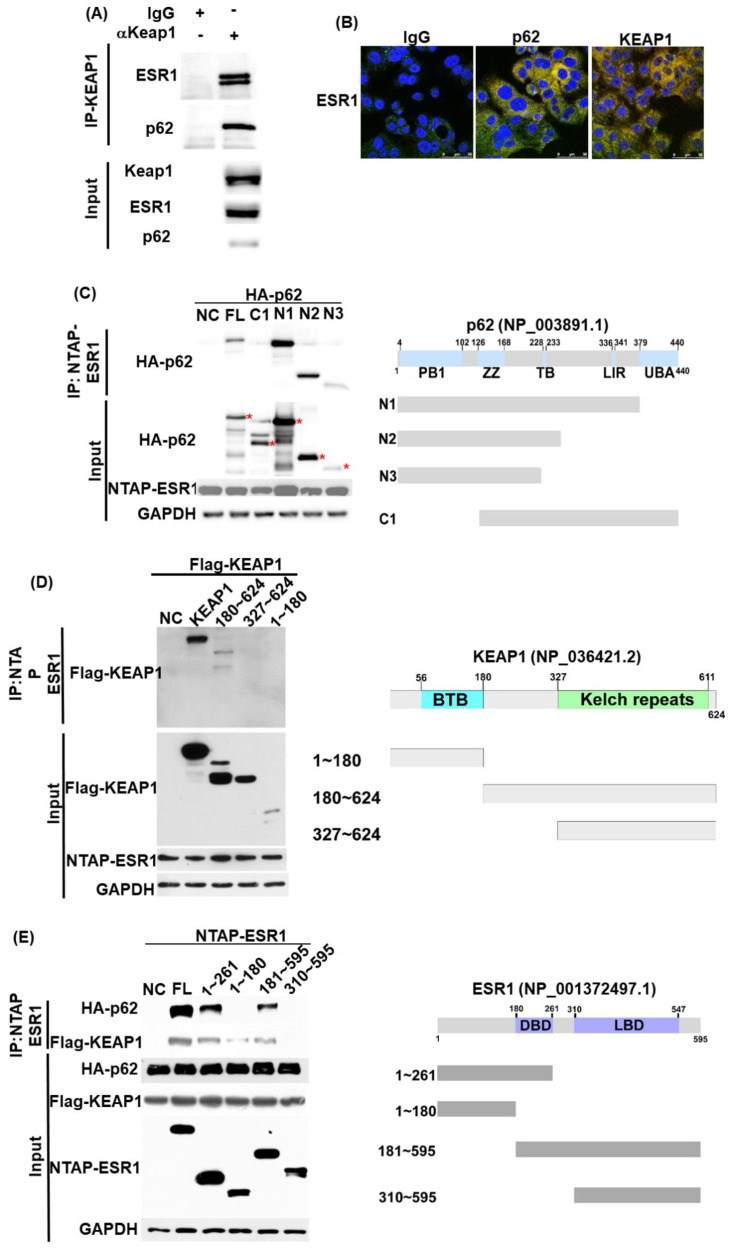
Estrogen receptor α (ESR1), p62, and KEAP1 are capable of forming a complex. (**A**) Using Ishikawa cell lysates, the endogenous complex containing KEAP1 was pulled down with an anti-KEAP1 antibody and a control IgG. Associated proteins—including ESR1 and p62—were identified with indicated antibodies. Expression levels of endogenous KEAP1, ESR1 and p62 are shown in the lower panel. (**B**) Confocal microscopy revealed the presence of an ESR1-p62–KEAP1 complex in Ishikawa cells. Cells were double stained with antibodies raised against ESR1 (green signal) plus control IgG (red signal, left panel), p62 (red signal, central panel) or KEAP1 (red signal, left panel). Overlapping green and red signals revealed the colocalization of two proteins as denoted by the yellow signal. (**C**) Two hundred and ninety-three cells were transfected with full-length ESR1 (NTAP-ESR1) and different truncated p62 constructs (N1, N2, N3, C1; HA tag). PB1, Phox and Bem1p-1; ZZ, zinc finger domain; TB, tumor necrosis factor-associated factor 6 binding motif; LIR, LC3 interaction region; UBA, ubiquitin-associated domain. The presence of ESR1 within a complex was examined with an anti-HA tag antibody. The asterisks indicate truncated p62 proteins identified in pulled-down complexes. (**D**) The full-length ESR1 (NATP-ESR1) was co-precipitated with full-length KEAP1 and three different truncated KEAP1 (FLAG-KEAP1) constructs (1−180; 180−624; 327−624). BTB: broad complex, tramtrack, and bric-à-brac. (**E**) Full-length p62 (HA-p62) or full-length KEAP1 (FLAG-KEAP1) were co-transfected with full-length ESR1 and four different truncated ESR1 constructs (1−261; 1−180, 181−595; and 310−595) in 293 cells. Following purification with streptavidin beads (NTAP-ESR1 constructs), coprecipitated p62 or KEAP1 were identified using an anti-HA or a FLAG antibody. DBD: DNA binding domain; LBD, ligand binding domain.

The interactions between the ESR1, KEAP1, and p62 proteins were initially investigated with immunoprecipitation. Using an anti-KEAP1 antibody in pull-down experiments, ESR1 and p62 were identified as being part of a complex that comprised KEAP1 (Figure 1A). Confocal microscopy further confirmed the colocalization of these proteins (Figure 1B). To shed light on the role played by specific domains in the reciprocal protein interactions, immunoprecipitation experiments with various truncated constructs were performed. We identified the PB1 domain of p62 (1−126; Figure 1C) and the central region of KEAP1 (180−327; Figure 1D) as responsible for the interactions with ESR1. Notably, the deleted DNA binding domain (DBD) in ESR1 (180−261) was found to decrease the interaction of ESR1 with both p62 and KEAP1 (Figure 1E). Collectively, these results indicate that ESR1, KEAP1, and p62 are capable of forming an intracellular complex—in which the DBD domain of ESR1, the central region of KEAP1, and the PB1 domain of p62 play a critical role. 

### 3.2. p62 Increases ESR1 Protein Stability

**Figure 2:**
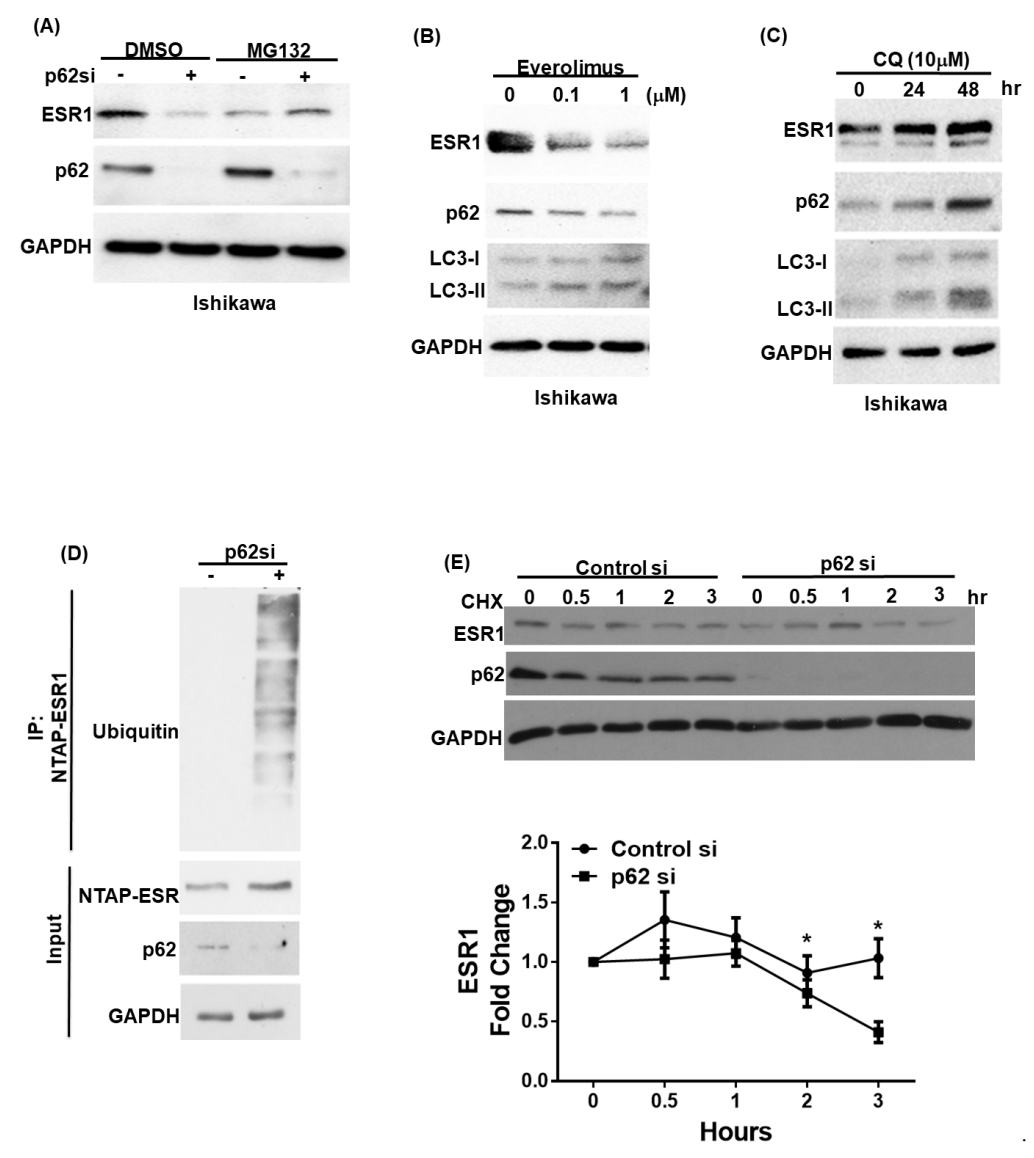
p62 and estrogen receptor α(ESR1) expression levels are reciprocally interrelated. (**A**) Ishikawa cells were transfected with a control vector or p62 siRNA for 72 h. Subsequently, they were harvested after exposure to either DMSO or a proteasome inhibitor (MG132; concentration: 10 μM) for 5 h. Detection of endogenous ESR1, p62, and GAPDH was performed using specific antibodies. Ishikawa cells were treated with either (**B**) the mTOR inhibitor everolimus to promote p62 degradation or (**C**) the autophagy inhibitor chloroquine (CQ) to induce p62 accumulation. ESR1, p62, LC3 and GAPDH were detected by Western blot. (**D**) NTPA-ESR1 (CBP tag) was transfected into 293 cells with and without silenced p62 siRNA expression and harvested after treatment with DMSO or MG132 (10 μM) for 5 h. NTAP-ESR1 was precipitated with streptavidin beads, whereas ubiquitin-labeled proteins were identified using an anti-ubiquitin antibody. Anti-CBP tag and GAPDH served as control inputs. An antibody raised against p62 was used to confirm the efficiency of p62 silencing. (**E**) Ishikawa cells with and without silenced p62 were harvested at the reported time points in presence of the protein synthesis inhibitor cycloheximide (CHX). Detection of endogenous ESR1 and p62 was performed using specific antibodies. GAPDH served as the loading control followed by normalization for ESR1 levels. The fold changes in ESR1 protein levels are reported in the lower panel. Results are expressed as means ± standard errors of the mean from three independent experiments; * *p* < 0.05.

We subsequently examined whether p62 can regulate ESR1 protein stability. To this aim, we initially investigated ESR1 protein levels in primary endometrial stromal cells, following p62 silencing. The knockdown of p62 was paralleled by a reduced ESR1 protein expression (Figure S1A) that was not accompanied by decreased ESR1 mRNA levels (Figure S1B). The reduction in ESR1 protein expression following p62 silencing was abrogated by the treatment of cells with the proteasome inhibitor MG132 (Figure 2A; Figure S1C). Since autophagy plays a key role in regulating p62 protein levels, we investigated how the activation or inhibition of autophagy modified ESR1 expression levels. As expected, the autophagy activator everolimus was found to promote p62 degradation in both Ishikawa cells and primary endometrial stroma cells. Notably, ESR1 expression was found to decrease in parallel (Figure 2B; Figure S1D). Conversely, the autophagy inhibitor chloroquine induced p62 protein accumulation and increased ESR1 expression (Figure 2C; Figure S1E). To further investigate the mechanisms by which p62 increases ESR1 protein levels, we assessed the presence of ubiquitin–ESR1 complexes in cells with and without silenced p62 expression. Compared with the control cells, those with silenced p62 expression showed higher levels of ubiquitin-labeled ESR1 (Figure 2D). Additionally, the turnover rate of ESR1 in cells with silenced p62 expression was accelerated compared with that observed in control cells treated with the protein synthesis inhibitor cycloheximide (Figure 2E). Collectively, these results demonstrate that p62 expression increases ESR1 protein stability.

### 3.3. GPR30 Activation by 17β-Estradiol Promotes p62 Phosphorylation and ESR1 Expression

**Figure 3:**
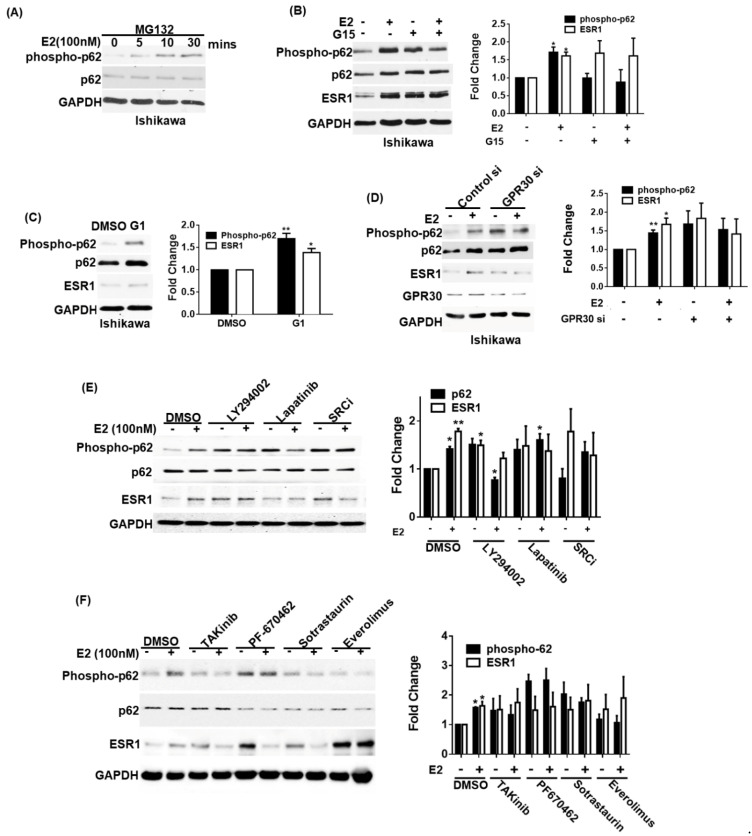
Activation of GPR30 by 17β-estradiol promotes p62 phosphorylation and estrogen receptor α (ESR1 expression)**.** (**A**) Ishikawa cells were pretreated overnight with the proteasome inhibitor MG132 (concentration: 5 μM) in phenol red-free OPTI-MEM medium. After harvesting, cells were exposed to 17β-estradiol (100 nM) at the reported time points. Phosphorylated and total p62 were detected using specific antibodies; endogenous GAPDH served as a loading control. (**B**) A pharmacological GPR30 antagonist (G15) inhibited 17β-estradiol-induced p62 phosphorylation and ESR1 expression. Ishikawa cells were starved overnight in presence of the proteasome inhibitor MG132 (concentration: 5 μM) in phenol red-free OPTI-MEM medium. On the next day, cells were pretreated with either G15 (100 nM) or a vehicle for 30 min and subsequently exposed to 17β-estradiol (100 nM) or a vehicle for 30 min. Protein levels of phosphorylated and total p62, ESR1, and GAPDH were examined with Western blot. Results for phosphorylated p62 and ESR1 were normalized for endogenous p62 and GAPDH expression levels. The fold changes for phosphorylated p62 and ESR1 protein levels are reported in the right panel. (**C**) A pharmacological GPR30 agonist (G1) enhanced p62 phosphorylation and ESR1 expression. Ishikawa cells were starved overnight in presence of the proteasome inhibitor MG132 (concentration: 5 μM) in phenol red-free OPTI-MEM medium and subsequently treated with G1 (10 nM). Protein levels of phosphorylated and total p62, ESR1, and GAPDH were examined with Western blot. Results for phosphorylated p62 and ESR1 were normalized for endogenous p62 and GAPDH expression levels. The fold changes for phosphorylated p62 and ESR1 protein levels are reported in the right panel. (**D**) Ishikawa cells were transfected with control or GPR30 siRNA for 72 h and subsequently exposed to 17β-estradiol (100 nM) or a vehicle for 30 min. Protein levels of phosphorylated and total p62, ESR1, and GAPDH were examined with Western blot. Results for phosphorylated p62 and ESR1 were normalized for endogenous p62 and GAPDH expression levels. The fold changes for phosphorylated p62 and ESR1 protein levels are reported in the left panel. (**E**,**F**) GPR30 downstream signaling pathways involved in p62 phosphorylation and ESR1 expression. Ishikawa cells were starved overnight in presence of the proteasome inhibitor MG132 (concentration: 5 μM) in phenol red-free OPTI-MEM medium and subsequently treated for 30 min with the following compounds: a PI3K inhibitor (LY294002), an EGF receptor inhibitor (lapatinib), a SRC inhibitor (SRCi), a TAK inhibitor (takinib), a casein kinase inhibitor (PF-670462), a PKC inhibitor (sotrastaurin), and an mTOR inhibitor (everolimus). Protein levels of phosphorylated and total p62, ESR1, and GAPDH were examined with Western blot. Results for phosphorylated p62 and ESR1 were normalized for endogenous p62 and GAPDH expression levels. The protein levels in vehicle control absence of E2 treatment as one. Results are expressed as mean ± standard errors from three independent experiments. Statistical significance was calculated with Student’s *t*-test * *p* < 0.05. ** *p* < 0.01.

It has been previously shown that phosphorylated p62 can bind to KEAP1 to stabilize NRF2 [12]. Therefore, we investigated whether ESR1 expression can be regulated by the p62–KEAP1 complex. We initially observed that exposure to 17β-estradiol promoted p62 phosphorylation in a time-dependent fashion—being evident after an exposure of at least 5 min (Figure 3A). We subsequently used a GPR30 antagonist (G15) and a GPR30 agonist (G1) to investigate the role played by GPR30 activation in 17β-estradiol-induced p62 phosphorylation and ESR1 expression. The pretreatment of Ishikawa cells with G15 inhibited 17β-estradiol-induced p62 phosphorylation and ESR1 expression (Figure 3B); conversely, G1 was able to directly induce these effects (Figure 3C). For further confirmation, we silenced GPR30 expression in Ishikawa cells. GPR30 silencing inhibited 17β-estradiol-induced p62 phosphorylation and ESR1 expression (Figure 3D). The activation of GPR30 by 17β-estradiol has been shown to elicit the SRC/EGFR/PI3K/Akt/mTOR pathway [4]. Since several kinases have been reported to phosphorylate p62 at serine 349, including protein kinase C (PKC) [20], casein kinase 1 [21], mTORC1 [12], and TAK1 [22], we analyzed the effects of specific kinase inhibitors aimed at targeting this pathway on 17β-estradiol-induced p62 phosphorylation and ESR1 expression. We found that a SRC inhibitor, lapatinib (an EGFR inhibitor), LY294002 (a PI3K inhibitor), TAKinib (a TAK inhibitor), PF-670462 (a casein kinase inhibitor), sotrastaurin (a PKC inhibitor), and everolimus (an mTOR inhibitor) successfully inhibited 17β-estradiol-induced p62 phosphorylation and ESR1 expression (Figure 3E,F). Collectively, these data indicate that the SRC/EGFR/PI3K/mTOR pathway is involved in the induction of p62 phosphorylation and ESR1 expression elicited by 17β-estradiol through GPR30 activation.

### 3.4. Phosphorylated p62 Binds to KEAP1 and Promotes the Release of ESR1 from a Protein Complex to Increase Its Expression

**Figure 4:**
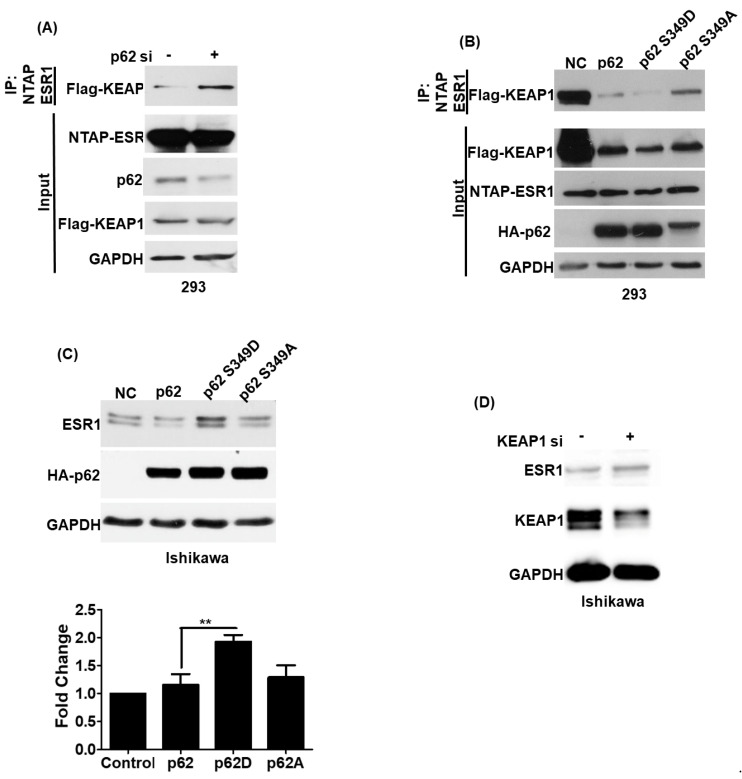
The KEAP1–p62 complex regulates estrogen receptor α (ESR1) protein expression. (**A**) NTAP-ESR1 and FLAG-KEAP1 were overexpressed in 293 cells either with or without p62 silencing. Following purification with streptavidin (NTAP-ESR1 construct), co-precipitated KEAP1 was detected using a FLAG antibody. (**B**) Two hundred and ninety-three cells were transfected with NTPA-ESR1, FLAG-KEAP1, and different HA-p62 constructs —including wild-type p62, the p62 S349D mutant (which mimicked the biological action of phosphorylated p62), and the p62 S349A mutant (which was unable to undergo phosphorylation). KEAP1–ESR1 complexes were identified using an anti-FLAG antibody. (**C**) HA-p62 (wild type), HA-p62 S349D, and HA-p62 S349A were overexpressed in Ishikawa cells. Subsequently, ESR1 and p62 protein levels were assessed using anti-ESR1 and HA antibodies, respectively. GAPDH served as loading control. The fold changes in ESR1 protein levels are reported in the bottom panel. Results are expressed as means ± standard errors of the mean from three independent experiments; ** *p* < 0.01. (**D**) KEAP1 silencing induced ESR1 expression in Ishikawa cells. Protein levels of ESR1, KEAP1, and GAPDH were examined with Western blot.

Previous research demonstrated that phosphorylated p62 can promote the release of NRF2 by interacting with KEAP1 [13]. Therefore, we analyzed whether phosphorylated p62 (S349) can promote the release of ESR1 from KEAP1–ESR1 complexes. We initially observed that p62 silencing led to an increased formation of the ESR1–KEAP1 complex (Figure 4A). To investigate whether phosphorylated p62 (S349) was able to increase ESR1 protein expression, two different p62 mutants affecting this critical amino acid residue were constructed. The p62 S349D mutant mimicked the biological action of phosphorylated p62, whereas p62 S349A was unable to undergo phosphorylation. While cells expressing the p62 S349D mutant showed a weaker ESR1–KEAP1 interaction, the opposite was evident for cells expressing the p62 S349A mutant (Figure 4B). We also observed that cells expressing the p62 S349D mutant had higher ESR1 protein levels compared with both cells expressing the p62 S349A mutant and wild-type cells (Figure 4C). KEAP1 silencing in Ishikawa cells was also found to increase ESR1 protein expression. Collectively, these results indicate that phosphorylated p62 binds to KEAP1 and promotes the release of ESR1 from the protein complex—ultimately increasing its protein expression.

### 3.5. 17β-Estradiol-Induced Cell Proliferation Is Inhibited by p62 Silencing

**Figure 5:**
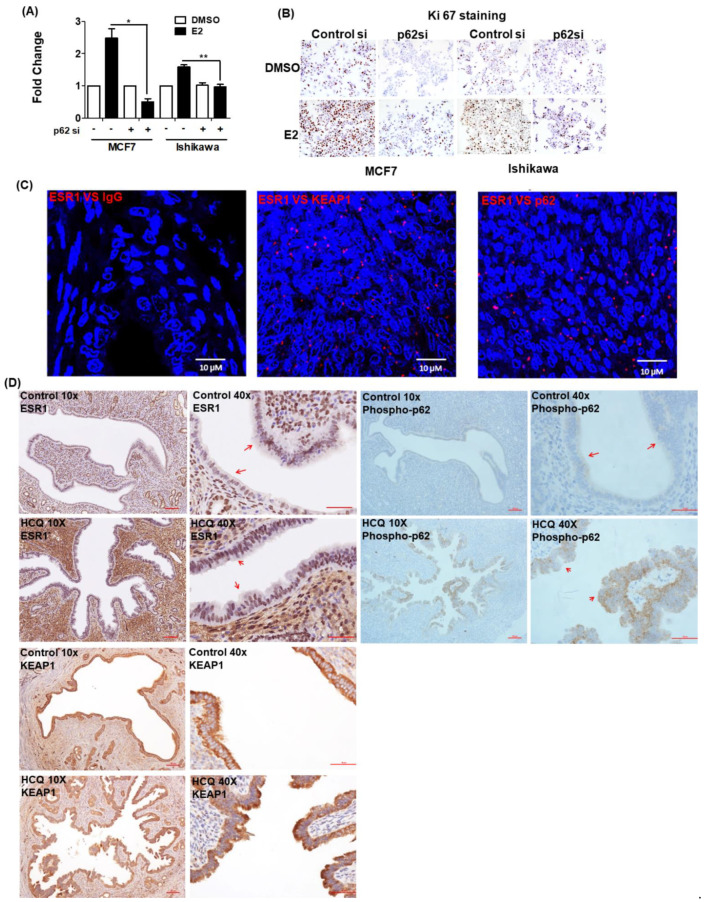
Phosphorylated p62 promotes estrogen receptor α (ESR1) expression. The results of (**A**) the BrdU assay and (**B**) Ki-67 staining revealed that the proliferation capacity of cells with silenced p62 expression was lower than that of control cells. MCF7 and Ishikawa cells were transfected for 48 h with control siRNA or p62 siRNA and subsequently exposed to 17β-estradiol (100 nM) or a vehicle for 24 h. Results are expressed as means ± standard errors of the mean from three independent experiments. * *p* < 0.05, ** *p* < 0.01. (**C**) The results of proximity ligation assay confirmed the interactions between ESR1, KEAP1, and p62 in human endometrial specimens. Staining was performed with antibodies raised against ESR1, KEAP1, and p62, with IgG serving as negative control. The red dots indicate protein interactions. The nuclei were stained with DAPI, which showed blue color. (**D**) Immunohistochemical staining of ESR1 (left panel), phosphorylated p62 (right panel) and KEAP1 (lower panel) in uterine epithelial cells of mice fed with a vehicle (upper panel) or hydroxychloroquine (HCQ, 50 mg/kg/day, lower panel) five days a week (magnifications: 10× and 40×). The arrowheads indicate different expression levels of ESR1 and phosphorylated p62.

17β-estradiol is known to promote the proliferation of estrogen-sensitive cells. Therefore, we investigated the role played by p62 expression in mediating this phenomenon. To this aim, we used the BrdU assay and Ki67 staining to investigate the proliferative capacity of MCF7 and Ishikawa cells, either with or without silenced p62 expression, after exposure to 17β-estradiol. Compared with control cells, p62 silencing inhibited 17β-estradiol-induced cell proliferation (Figure 5A). Ki67 staining confirmed a lower proliferation capacity of cells with silenced p62 expression after 17β-estradiol exposure (Figure 5B). Collectively, these results indicate that p62 plays a key role in mediating 17β-estradiol-induced cell proliferation.

### 3.6. ESR1 Interacts with Both KEAP1 and p62 In Vivo

PLA was used to examine the interactions between ESR1 and the KEAP1–p62 complex in formalin-fixed paraffin-embedded human endometrial tissue. The findings confirmed that ESR1 interacted with both KEAP1 and p62 (Figure 5C). The results of animal experiments revealed that treatment with hydroxychloroquine—an analogue of chloroquine with less toxic in vivo effects compared with chloroquine [23]—for one month, promoted the expression of both ESR1 and phosphorylated p62 in uterine epithelial cells; however, KEAP1 expression was not affected (Figure 5D). Collectively, these results provide evidence that the KEAP1–p62 complex can regulate ESR1 expression in vivo.

## 4. Discussion

**Figure 6:**
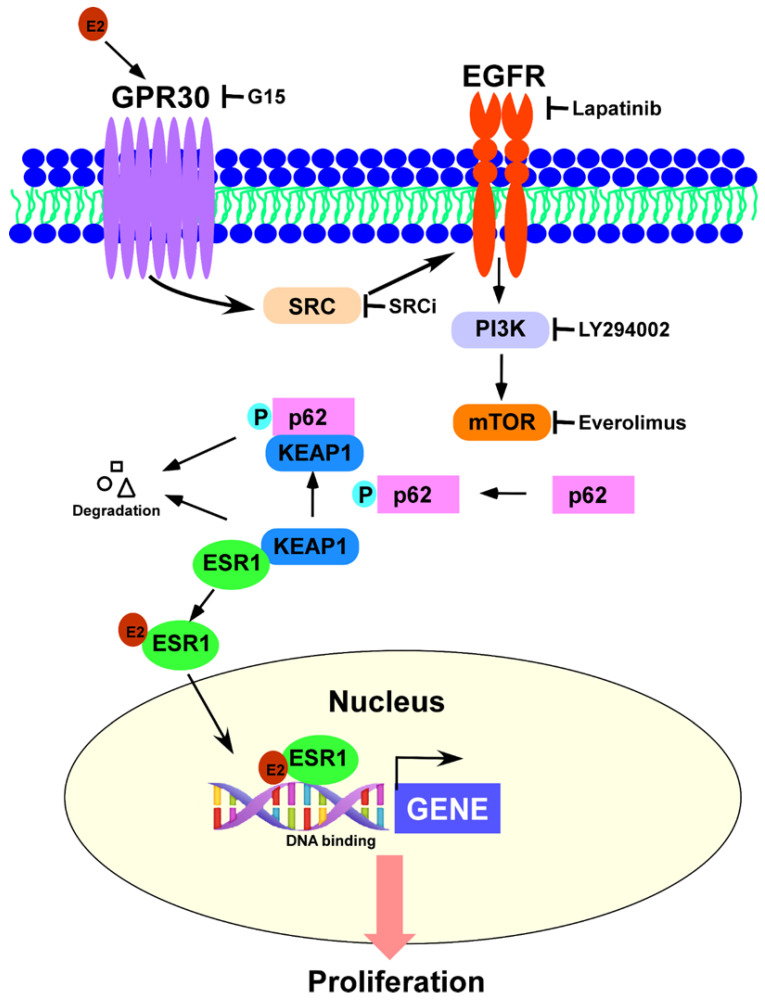
GPR30 activation by 17β-estradiol elicits the SRC/EGFR/PI3K/Akt/mTOR signaling pathway and promotes p62 phosphorylation—which in turn induces ESR1 protein expression through estrogen receptor α (ESR1) detachment from a complex formed with KEAP1. This complex interplay provides a link between the rapid-signal effects of estrogens (mediated by GRP30) and their canonical genomic effects (mediated by ESR1).

The results of our study indicate that GPR30 activation by 17β-estradiol elicits the SRC/EGFR/PI3K/Akt/mTOR signaling pathway and promotes p62 phosphorylation—which, in turn, induces ESR1 protein expression through a detachment mechanism from a complex formed with KEAP1 (Figure 6). This intricate interplay provides a link between the rapid effects of estrogens (mediated by GRP30) and their canonical genomic effects (mediated by ESR1). While ESR1 has been previously shown to promote GPR30 expression in the endometrial epithelium [24], our findings demonstrate, for the first time, that signaling by 17β-estradiol can ultimately influence canonical genomic responses to estrogens, by promoting ESR1 expression through p62-mediated mechanisms. These findings may have significant therapeutic implications. While most of the current endocrine therapies that block estrogen signaling target ESR1, our results suggest that blockade of GPR30-mediated non-canonical estrogen receptor signaling can also ultimately affect ESR1 expression. When GPR30-mediated signaling was inhibited, ESR1 downregulation was observed. Collectively, these findings may pave the way to a combinatorial or complementary strategy for inhibiting estrogen signaling—which can complement the existing therapeutics to improve the clinical outcomes of endocrine therapies in estrogen-sensitive cancers.

One of the key observations in our study is that the activation of GPR30 signaling promoted p62 phosphorylation and increased ESR1 protein expression by inducing its release from a complex formed with KEAP1. When p62 is unphosphorylated, the levels of ESR1 are kept low by a complex with KEAP1. In these conditions, KEAP1 ubiquitinates the ESR1 protein and targets it for proteasomal degradation—a mechanism that has been previously reported for the KEAP-1-mediated regulation of the transcription factor NRF2 [25]. Therefore, in the absence of GPR30 signaling, a constitutive degradation of ESR1 allows for only the basal expression of the receptor. However, GPR30 activation by 17β-estradiol promotes p62 phosphorylation—which competes with ESR1 for binding with KEAP1, and ultimately elicits its dissociation from the complex. This mechanism—which appears to be similar to that described for the KEAP1–NRF2 interaction [25]—has not been previously reported for ESR1. It is interesting to note that either p62 silencing or the use of a mutant p62 (S349A) that was unable to undergo phosphorylation led to increased formation of the ESR1–KEAP1 complex, ultimately reducing ESR1 expression. Based on the results of immunoprecipitation with different ESR1, KEAP1, and p62 constructs, we speculate that phosphorylated p62 may induce conformational changes in the central region of KEAP1 —which, in turn, weakened the interaction with ESR1.

Estrogen signaling is involved in the crosstalk of autophagic pathways. However, the role of estrogen to promote or inhibit autophagy is still debatable [26]. In our results, estrogen stimulates membrane ESR1 or GPR30, activating the PI3K-mTOR pathway that may block autophagy. Meanwhile, KEAP1-NRF2 signaling is also involved in the mTOR pathway, and regulates autophagy [27,28]. In circumstances of impaired autophagy, for instance, the accumulation of p62 promotes bladder cancer cell proliferation via sequestrating *KEAP1* to upregulate antioxidant genes and protect cancer cells from oxidative stress [29]. In another scenario, somatic mutations of *KEAP1* identified in lung cancer patients promote tumorigenesis [30].

Since p62 may operate as an adaptor protein to transport misfolded proteins for autophagic degradation, we also examined how this process affected ESR1 expression. The activation of autophagy by everolimus decreased ESR1 by promoting p62 degradation, whereas autophagy inhibition with chloroquine increased ESR1 expression. As autophagy appears at the cross-road of both rapid signaling and canonical effects of estrogens, the application of autophagy activators and inhibitors warrants additional investigations in various estrogen-dependent clinical conditions. While autophagy activation by everolimus may improve the clinical outcomes of women with estrogen-dependent malignancies, via decreased ESR1 expression [31], more research is needed to understand the clinical implications of our findings and how different estrogen signaling modalities integrate at the level of gene transcription.

## 5. Conclusions

The results of our study indicate that 17β-estradiol-mediated GPR30 activation elicits the SRC/EGFR/PI3K/mTOR signaling pathway to phosphorylate p62. In turn, phosphorylated p62 promotes the release of ESR1 from complexes with KEAP1—ultimately increasing its protein expression. Our findings shed light on the molecular underpinning of 17β-estradiol signaling through GPR30, and may lead to novel signaling- or autophagy-mediated pharmacological strategies aimed at decreasing ESR1 expression in estrogen-sensitive cells.

## Data Availability

The data used in the present study are available from the corresponding author upon reasonable request.

## Supplementary Materials

The following are available online at https://www.mdpi.com/article/10.3390/jpm11090906/s1, Figure S1: p62 promotes ESR1 expression in primary endometrial stromal cells. Table S1: Sequences of primers used in the study.
