# Supplementary material for: GPR30 Activation by 17β-Estradiol Promotes p62 Phosphorylation and Increases Estrogen Receptor α Protein Expression by Inducing Its Release from a Complex Formed with KEAP1
Source: J Pers Med. 2021 Sep 11;11(9):906. doi: 10.3390/jpm11090906 (PMC8468056; doi:10.3390/jpm11090906)
Supplement: Supplementary file 1 [file jpm-11-00906-s001.zip › jpm-1286615-supplementary.pdf]

Supplementary information

GPR30 activation by 17β-estradiol promotes p62 phosphorylation and increases estrogen receptor α protein expression by inducing its release from a complex formed with KEAP1

Chia-Lung Tsai[1,†], Chiao-Yun Lin[2,3, †], Angel Chao[2,3], Yun-Shien Lee[1,4], Ren-Chin Wu[5], Chi-Neu Tsai[6], Chih-Feng Yen[2], An-Shine Chao[2,7,*]

[1]Genomic Medicine Research Core Laboratory, Chang Gung Memorial Hospital, Taoyuan, Taiwan

[2]Department of Obstetrics and Gynecology, Linkou Chang Gung Memorial Hospital and Chang Gung University College of Medicine, Taoyuan, Taiwan

[3]Gynecologic Cancer Research Center, Linkou Chang Gung Memorial Hospital, Taoyuan, Taiwan

[4]Department of Biotechnology, Ming-Chuan University, Taoyuan, Taiwan

[5]Department of Pathology, Linkou Chang Gung Memorial Hospital, Taoyuan, Taiwan

[6]Chang Gung University College of Medicine, Taoyuan, Taiwan

[7]Department of Obstetrics and Gynecology, New Taipei Municipal Tu Cheng Hospital, New Taipei City, Taiwan

†These authors contributed equally to this work.

*Correspondence: An-Shine Chao, MD, Department of Obstetrics and Gynecology New Taipei City Municipal Tu Cheng Hospital, New Taipei City, Taiwan

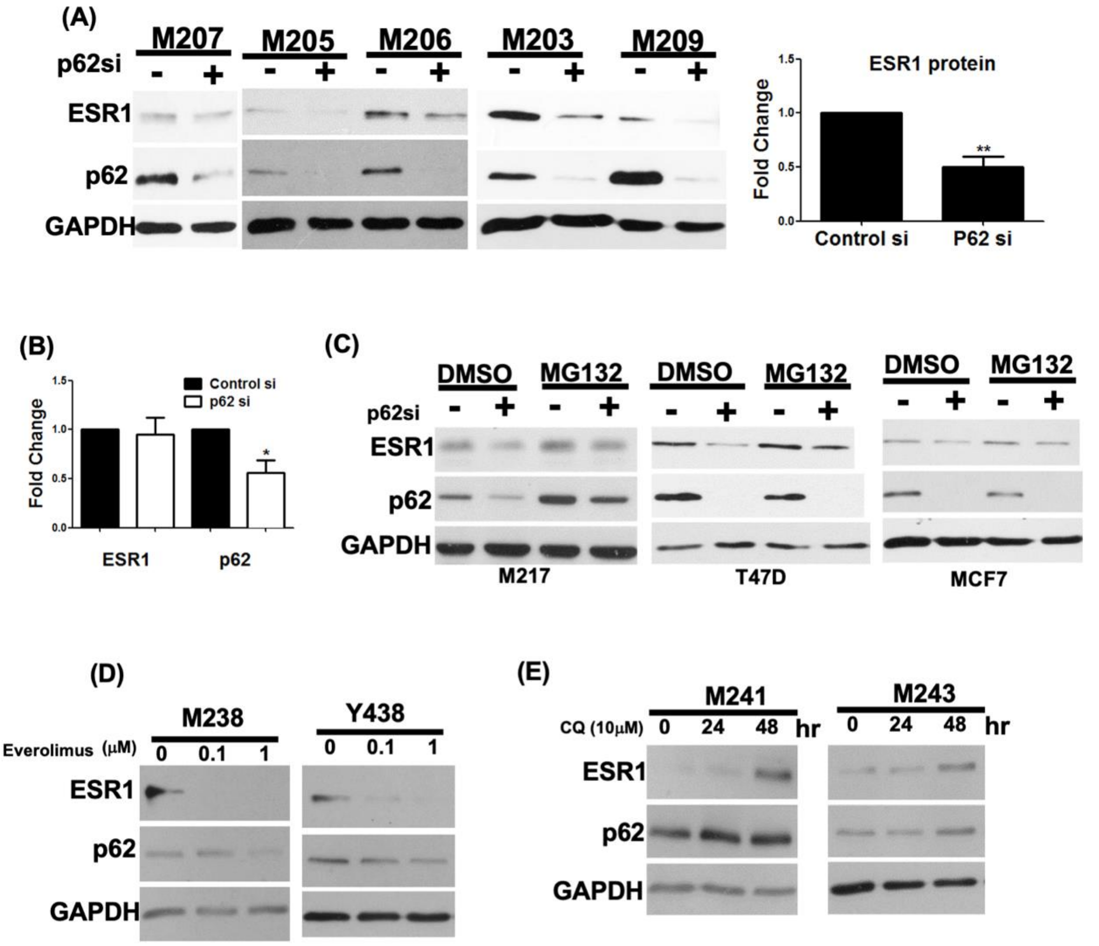

Figure S1. p62 promotes ESR1 expression in primary endometrial stromal cells. **(A)** Protein expression levels of ESR1, p62, and GAPDH were examined with western blot in five different primary endometrial stroma cell lines either in presence or absence of silenced p62 expression. The fold changes in ESR1 protein levels in presence *versus* absence of silenced p62 expression are reported in the right panel. **(B)** Fold changes in ESR1 and p62 mRNA levels were examined with qPCR in primary endometrial stromal cells in presence *versus* absence of silenced p62 expression (control conditions set at 1). **(C)** Primary endometrial stroma cell (left panel) and breast cancer cells (T47D and MCF7, middle and right panel) were treated with DMSO or the proteasome inhibitor MG132 (10 mM) for 5 h either in presence or

absence of silenced p62 expression. Protein levels of ESR1, p62, and GAPDH were examined with western blot. Primary endometrial stroma cell lines were treated at different time points with the reported concentrations of an autophagy activator (everolimus) **(D)** or an autophagy inhibitor **(**chloroquine; CQ) **(E)** to promote the degradation or induce the accumulation of p62, respectively. Protein levels of ESR1, p62, and GAPDH were examined with western blot.

Table S1. Sequences of primers used in the study

Primer	Sequence (5’-3’)
P62 S349A F	AGTGGACCCGGCTACAGGTGA
P62 S349A R	TCTTTTGAAGACAGATGGGTCCAG
P62 S349D F	AGTGGACCCGGATACAGGTGAAC
P62 S349D R	TCTTTTGAAGACAGATGGG
Keap1 3xFlag F	TAGCCCGGGCGGATCCATGCAGCCAGATCCCAGGC
Keap1 3xFlag R	ATCGATAAGCTTGATATCACAGGTACAGTTCTGCTGGTCAATC
Keap1 180 R	ATCGATAAGCTTGATATCCTGCTGCACCAGGAAGTCAC
Keap1 180 F	TAGCCCGGGCGGATCCCTGGACCCCAGCAATGCC
Keap1 327 F	TAGCCCGGGCGGATCCCCCAAGGTGGGCCGCCTG
NTAP-ESR1 F	CTGCCCGGGCGGATCCATGACCATGACCCTCCACAC
NTAP-ESR1 R	GCTTGATATCGAATTCTCAGACCGTGGCAGGGA
ESR1 261R	GCTTGATATCGAATTCTCCTCTTCGGTCTTTTCGTATC
ESR1 180R	GCTTGATATCGAATTCGTAGCGAGTCTCCTTGGCAG
ESR1 181F	CTGCCCGGGCGGATCCTGTGCAGTGTGCAATGACTATG
ESR1 310F	CTGCCCGGGCGGATCCCTGACGGCCGACCAGAT
